# Desformylflustrabromine (dFBr), a positive allosteric modulator of α_4_β_2_ nicotinic acetylcholine receptors decreases voluntary ethanol consumption and preference in male and female Sprague-Dawley rats

**DOI:** 10.1371/journal.pone.0273715

**Published:** 2022-09-09

**Authors:** Steven Decker, Gregory Davis, Imran Vahora, Alen Vukovic, Parth Patel, Asha Suryanarayanan

**Affiliations:** Department of Pharmaceutical Sciences, Philadelphia College of Pharmacy, University of the Sciences, Philadelphia, PA, United States of America; Weizmann Institute of Science, ISRAEL

## Abstract

Alcohol use disorder is a medical condition that impacts millions of individuals worldwide. Although there are a few pharmacotherapeutic options for alcohol-dependent individuals; there is a need for the development of novel and more effective therapeutic approaches. Alcohol and nicotine are commonly co-abused, and there is evidence that neuronal nicotinic acetylcholine receptors (nAChRs) play a role in both alcohol and nicotine dependence. Desformylflustrabromine (dFBr), a positive allosteric modulator of the α_4_β_2_ nAChRs has been shown to reduce nicotine intake, compulsive-like behavior and neuropathic pain in animal models. dFBr has also been previously shown to cross the blood-brain-barrier. We have recently shown that dFBr can attenuate the response to an acute, hypnotic dose of ethanol, via β_2_ nAchR. Here, we have investigated the effect of dFBr in modulating ethanol consumption using the intermittent access two-bottle choice (IA2BC) model of voluntary ethanol consumption in male and female Sprague Dawley rats. We show that dFBr selectively reduced ethanol but not sucrose consumption in the IA2BC model. Furthermore, dFBr decreased preference for ethanol in both male and female rats. No rebound increase in ethanol intake was observed after the washout period after dFBr treatment. The ability of dFBr to decrease ethanol consumption, along with its previously demonstrated ability to decrease nicotine self-administration in rodents, suggest that dFBr is an attractive therapeutic candidate to target both nicotine and alcohol abuse.

## Introduction

According to the 2019 National Survey on Drug Use and Health (NSDUH), nearly 9 million people men and 5.5 million women ages 12 and older had alcohol use disorder (AUD) in the United States. However, only about 7.2 percent of such individuals who match the criteria for AUD received any treatment in the past year [1]. Although Food and Drug Administration (FDA) approved medications to treat AUD have shown efficacy, the effect sizes of these drugs are sub-optimal. Moreover, the COVID-19 pandemic is also associated with increased alcohol consumption [2]. Therefore, there is a need for new pharmacotherapies to target AUD [3,4].

Neuronal nicotinic acetylcholine receptors (nAChRs) belong to the cys-loop superfamily of ligand-gated ion channels and mediate cation influx upon activation by acetylcholine (ACh) or exogenous ligands such as nicotine [5]. The two most commonly found nAChR subtypes in the mammalian brain are the α_4_β_2_* heteromeric (* indicates possible presence of other subunits, henceforth referred as α_4_β_2_) and the α_7_ homomeric receptors [6]. Both alcohol and nicotine stimulate dopaminergic neurons in the ventral tegmental area (VTA), increase dopamine release in the nucleus accumbens (NAcc), leading to drug reinforcement [7–9]. Further supporting overlapping mechanisms between nicotine and alcohol, ethanol has been shown to modulate reward via α_4_ subunit-containing nAChR located in the VTA [10,11]. Moreover, ACh levels in the VTA and DA levels in the NAcc are increased in animals consuming ethanol [12]. We have recently shown that desformylflustrabromine (dFBr), a commercially available positive allosteric modulator (PAM) that is selective for α_4_β_2_, but not α_3_β_2_ or α_7_ nAChRs [13,14] attenuates the response to an acute hypnotic dose of ethanol [15]. Specifically, dFBr is a type II PAM, i.e. it possesses no intrinsic activity, but increases the activity of an agonist acting at the target receptor [16]. Thus, PAMs are thought to cause fewer side effects and lower degree of tolerance, since unlike agonists, a PAM enhances receptor activity without interfering with endogenous patterns of synaptic transmission. Importantly, dFBr crosses the blood-brain barrier and has a predicted half-life of ~ 8.6 hrs [17].Previous studies have also shown that dFBr has therapeutic potential in reducing nicotine self-administration [17], decreasing compulsive-like behavior [18] and decreasing neuropathic pain [19]. Thus dFBr may represent a novel treatment strategy for the treatment of alcohol and/or nicotine addiction, since both these substances modulate the reward pathway via nAChR containing the α_4_ subunit.

In this study, we hypothesized that dFBr would decrease ethanol drinking and preference in male and female Sprague Dawley (SD) rats. We adapted the IA2BC model of alcohol abuse, which has been previously employed as a useful model for alcohol abuse in outbred rats, wherein rats provided intermittent access to 20% ethanol escalate ethanol consumption of ~ 5–6 g/kg/24 hrs (reviewed in [20]). This non-operant model provides an efficient and simple method to train animals to voluntarily consume high, clinically relevant levels of ethanol, similar to levels observed in human alcoholics. The IA2BC model is thought to better model alcohol abuse, rather than alcohol dependence, and represents a useful preclinical model to evaluate compounds targeting alcohol abuse [20]. Here, we employed the IA2BC model to study the effects of dFBr on voluntary ethanol consumption in male and female SD rats [20].

## Materials and methods

### Animals

All animal handling procedures were approved by the Institutional Animal Care and Use Committee (IACUC) at University of the Sciences and were conducted according to NIH specifications outlined in the Guide for the care and use of laboratory animals. Adult male and female SD rats (PND 70) were obtained from Charles River and housed in an Assessment and Accreditation of Laboratory Animal Care (AAALAC, Frederick, MD USA)-accredited facility. Food and water were available *ad libitum*.

In order to measure ethanol/water/sucrose consumption by each individual rat, animals were singly housed. Standard enrichment devices (plastic tubes) provided by the vivarium were present in all cages. Sight and smell of other rats could be perceived by rats at all times since animal cages were placed on shelves with a spacing of <0.5 inch between cages.

### Chemicals

dFBr hydrochloride was obtained from Abcam (Cambridge, Massachusetts, United States). Ethanol was purchased from Pharmaco-Aaper (Brookfield, Connecticut, United States). Other salts and buffering agents were obtained from Sigma–Aldrich (St. Louis, Missouri, United States). For behavioral experiments, ethanol was diluted in 0.9% saline solution (Open field assay) or tap water (IA2BC test) to a final concentration of 20%. dFbr stock solutions were prepared in 0.9% saline.

### IA2BC test

**Fig 1:**
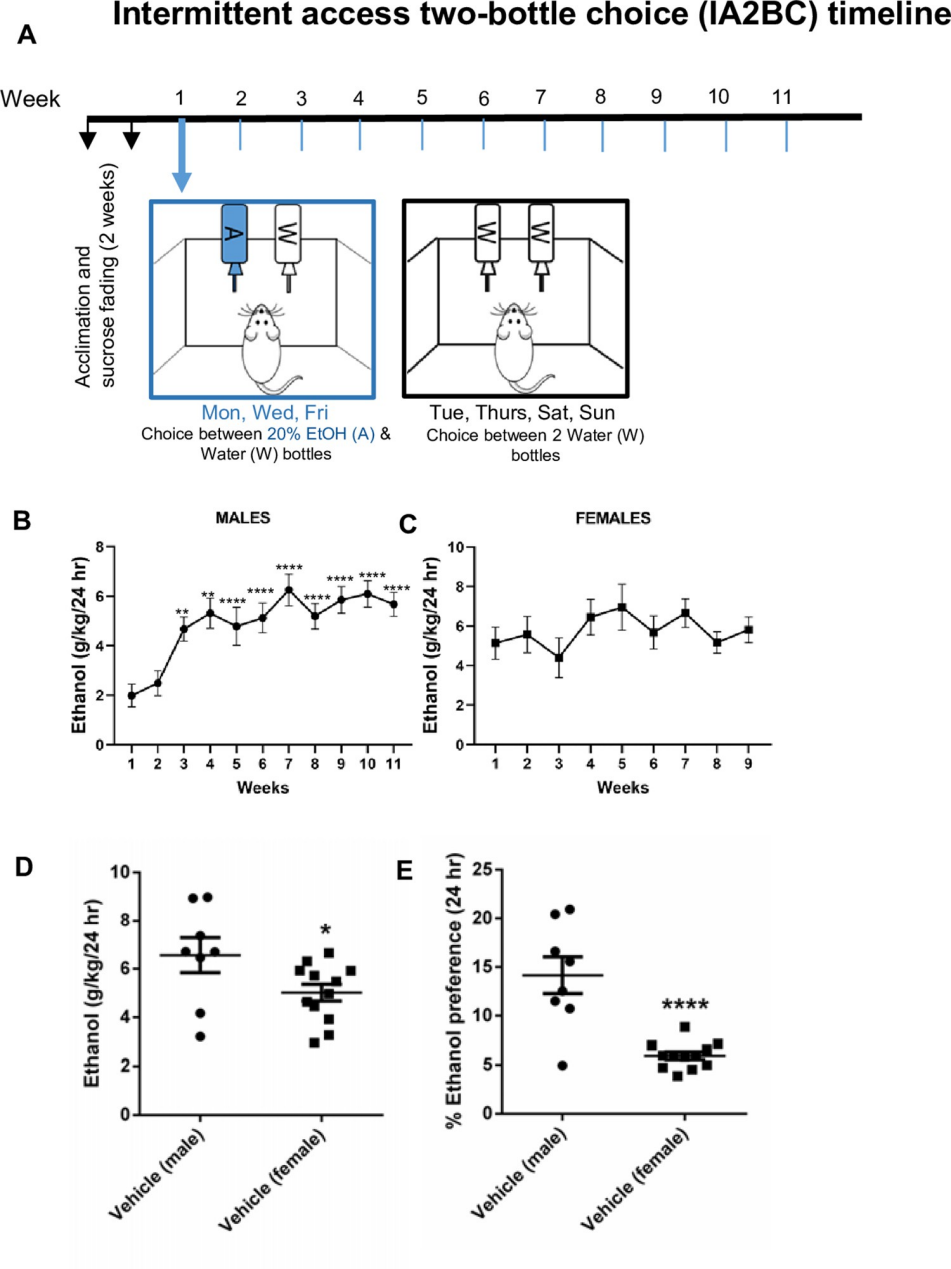
A. Establishment of an intermittent access two-bottle choice (IA2BC) model of voluntary alcohol consumption in adult male and female SD rats. Rats were provided access to 20% ethanol in tap water or water on Mon, Wed and Fri. Water was provided in both bottles on opposing days. This model was continued for 11 weeks (in males) and for 9 weeks (females), followed by 1 week of vehicle/dFBr injections. B. Ethanol drinking established in males (experiment 1+2, n = 23) is shown C. Ethanol drinking established in females (Experiment 2, n = 12) is shown. ** *p*<0.01, ***p< 0.0001 as compared to Week 1 drinking levels. D. Vehicle-treated male SD rats consumed significantly higher amount of ethanol (g/kg) as compared to vehicle-treated female rats (Experiment 2, n = 8 males/group, n = 12 females/group). E. Vehicle-treated male SD rats showed a higher preference for ethanol as compared to vehicle-treated female rats (Experiment 2, n = 8 males/group, n = 12 females/group). Data are represented as mean ± S.E.M. * *p*<0.05, ****p< 0.0001 compared to vehicle treatment group.

We adopted an IA2BC model described previously [20] and added a 2-week period of sucrose fading before switching the rats to 20% ethanol in tap water (Fig 1A). Upon arrival, animals were housed singly and acclimated to the home cage environment for one week. Rats were provided with 2 bottles in each cage. During week 1 after acclimation, one bottle had tap water and the other had 20% sucrose every other day resulting in sucrose access on Mon, Wed and Fri. In week 2, ethanol was introduced to sucrose solution at 5%. The ethanol was increased to 20% and sucrose was lowered to 0% over the course of 1 week. Following sucrose fading, rats were given access to 20% ethanol bottles on Mon, Wed, and Fri (Fig 1A). On the opposite days they had access to 2 bottles of tap water. The bottle’s location in the cage was changed every day to prevent any bias due to side-preference. The time of bottle changing was the same time each day. This schedule was maintained for 11 weeks (week 1–11 shown in Fig 1A), until rats escalated drinking and demonstrated stable ethanol drinking levels. Beginning at Week 1 of acclimation, fluid intake (the amount of ethanol or water consumed) was determined by weighing the bottles before access and after 24 hr of access to 20% ethanol and water. This choice was presented every Mon, Wed and Fri for the entire duration of the experiment. “Control” bottles with filled with ethanol/water were used at all times to estimate any possible leaks. Minimal leaks were noted in our experiments.

In experiment 1, male rats were studied and the amount of ethanol, water and food consumed was recorded each day at the 24 hour period, i.e. 24 hr after 20% ethanol/water presentation. (n = 15 each for vehicle, 1 mg/kg dFBr and 3 mg/kg dFBr treatment groups). Thus, 4 hr ethanol and water consumption was not recorded in experiment 1. We did not find any outliers that did not escalate drinking in this experiment. In the same experiment, we also employed another cohort of male rats exposed to intermittent exposure to 20% sucrose on Mon, Wed, Fri and water on other days (n = 8 each for vehicle, 1 mg/kg dFBr and 3 mg/kg dFBr treatment groups).

In experiment 2, we included both male (n = 10 each for vehicle, 1 mg/kg dFBr and 3 mg/kg dFBr treatment groups) and female (n = 12 each for vehicle, 1 mg/kg dFBr and 3 mg/kg dFBr treatment groups) rats. Ethanol and water intake were recorded at 4 hr as well as 24 hr after presentation of 20% ethanol and water bottles. Fluid intake was normalized to body weight of rat (g/kg). We excluded 2 male rats/group from the initial n = 10 males/group in this experiment since they did not significantly escalate drinking from Week 1–11. Food consumption by each rat was also recorded at the 24 hr time period. In both experiments, food was available *ad libitum* and body weight of all animals was monitored. Investigators administering the treatments and analyzing the data were blind to the treatments.

In both experiments, after reaching stable drinking levels (week 11), animals were randomized into 3 groups: Vehicle control, 1 mg/kg dFBr and 3 mg/kg dFBr. These doses were chosen based on previous rodent studies with dFBr examining nicotine self-administration [17], compulsive-like behavior [18] and our previous study examining the hypnotic dose of ethanol [15]. Each group received its corresponding treatment via subcutaneous (s.c.) injection on Mon, Wed and Fri, 1 hour prior to EtOH access. Thus, vehicle or dFBr treatment occurred in the IA2BC test via a total of 3 doses (Mon, Wed and Fri in week 11 of the timeline) after stable drinking levels were reached). Drinking data are presented as average weekly ethanol & water drinking levels (Average of Mon, Wed and Fri levels in each week of the IA2BC timeline). % preference for ethanol was calculated as follows:

$$\frac{\mathrm{ml}\;\mathrm{of}\;20\%\;\mathrm{ethanol}\;\mathrm{intake}}{\mathrm{ml}\;\mathrm{of}\;\mathrm{total}\;\mathrm{fluid}\;\mathrm{intake}\;(\mathrm{water}+20\%\;\mathrm{ethanol})}X\;100$$

### Open field assay

An open field (OF) assay was established to determine if dFBr administration affected locomotion in SD rats and if dFBr pretreatment altered locomotion in response to a low dose of ethanol (2 g/kg). A 2 X 2 feet arena with elevated walls was built for the open field assay. The floor and walls of the arena were covered, with the top left open. Adult SD rats were given varying treatments (Vehicle, 3.0 and 6.0 mg/kg dFBr s.c.), 30 minutes prior to receiving either saline or ethanol (2g/kg) via i.p. injection before being placed into the arena. Video was recorded for 30 minutes after placement into the arena. SMART software (Harvard Apparatus, Holliston, MA, United States) was used to analyze the videos to calculate total movement within the arena.

### Data analysis

Statistical analysis was performed by using Prism software (GraphPad, San Diego, CA, United States), and data were analyzed by Repeated measures (RM) One-way ANOVA or One-way ANOVA followed by Dunnett’s multiple comparisons test or Bonferroni multiple comparisons test when a significant overall main effect was found (*p* < 0.05) or by Student’s *t* test (unpaired) where appropriate. Data are represented as mean ± S.E.M.

## Results

### Male SD rats show higher voluntary ethanol consumption and preference in the IA2BC test as compared to female SD rats

Fig 1A shows the IA2BC paradigm employed. Fig 1B and 1C show the overall drinking pattern observed for male and female rats. We found that male rats escalated drinking to reach an average of ~6 g/kg/24 hr in the 11 week timeline. RM ANOVA found a significant difference between treatment weeks [F(3.533, 77.73) = 7.461, p<0.0001] in male rats. Specifically, ethanol drinking started escalating significantly Week 3 [p<0.01] and Week 5 [p<0.0001] onwards. In contrast, we found that female rats did not significantly escalate their ethanol drinking up to 9 weeks into the IA2BC timeline, but rather maintained an average of ~5g/kg/24 hr from week 1–9 (RM ANOVA found no significant difference between treatment weeks). Note that no dFBr was administered during this period of 9 weeks (females) or 11 weeks (males).

To compare male vs. female ethanol drinking and preference achieved in a comparable number of SD rats subjected to the IA2BC paradigm at the same time, we compared data from experiment 2 (n = 8 males/group, n = 12 females/group, note that 2 males/group were excluded from the initial n = 10 males/group planned for this experiment since these males did not significantly escalate ethanol drinking in 11 weeks). These excluded male rats displayed initial ethanol drinking levels of ~ 2g/kg/24 hr in Week 1 and did not show any significant escalation in Week 11 (unlike other male rats in this cohort). We found that female rats displayed lower ethanol intake (p<0.05) and preference for ethanol (p < 0.0001) as compared to male rats (Fig 1D and 1E).

### dFBr decreases voluntary ethanol consumption and preference in the IA2BC test in male and female SD rats

**Fig 2:**
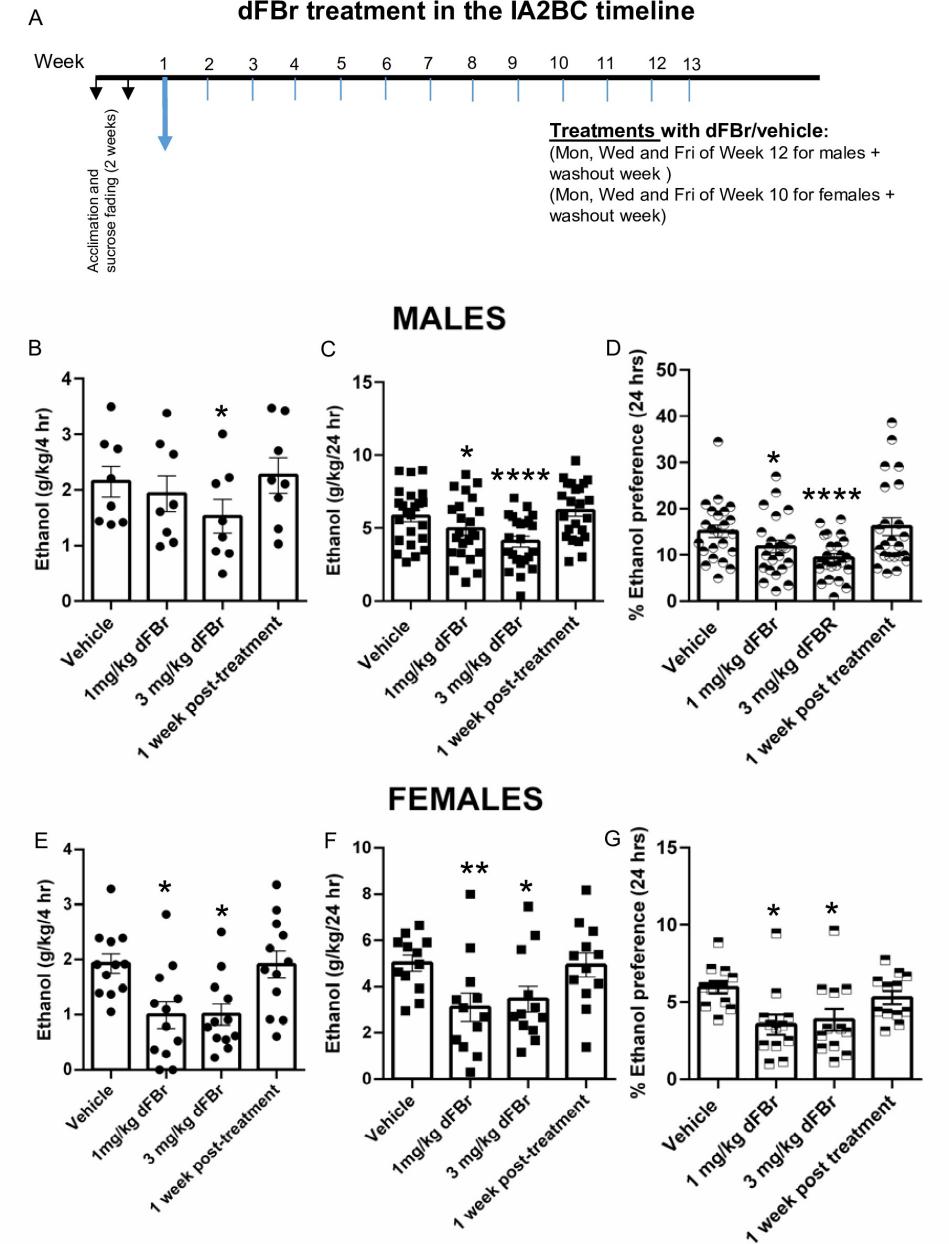
A shows the week of vehicle/dFBr injections dFBr in the IA2BC timeline followed by 1 week post-treatment washout period. dFBr significantly decreases ethanol consumption and preference for ethanol. Average drinking values/week are expressed as mean ethanol consumed (g/kg) ± S.E.M. dFBr (1 and 3 mg/kg s.c) or vehicle was administered 30 minutes prior to presentation of alcohol and water bottles via a total of 3 doses on Mon, Wed and Fri. B. dFBr significantly decreases voluntary 20% ethanol consumption at the 4 hr time-point in male SD rats, n = 8/group. C. dFBr significantly decreases voluntary 20% ethanol consumption at the 24 hr time-point in male SD rats (n = 23/group, Experiment 1+2). D. dFBr significantly decreases preference for ethanol at the 24 hr time-point in male SD rats (n = 23/group, Experiment 1+2). E. dFBr significantly decreases voluntary 20% ethanol consumption at the 4 hr time-point in female SD rats (n = 12/group). F. dFBr significantly decreases voluntary 20% ethanol consumption at the 24 hr time-point in female SD rats (n = 12/group). G. dFBr significantly decreases preference for ethanol at the 24 hour timepoint in female SD rats (n = 12/group). * *p*<0.05,**p<0.01, ****p<0.0001 compared to vehicle treatment group.

Fig 2 and Table 1 show data representing weekly average drinking values on Mon, Wed and Fri when ethanol and water bottles were presented (Fig 2, Table 1) as well as drinking values in the week when vehicle/dFBr was administered. Values from Experiment 1 and 2 have been pooled together for male rats for the 24 hr time-point since both experiments examined dFBr effects in male rats at the 24 hr time-point. Note that Experiment 2 examined the 4 hr as well as 24 hr timepoint in both male and female rats. At the 4 hour time-point after dFBr administration, ANOVA found a significant difference between treatment groups [F (2.055, 14.39) = 7.436, p< 0.0001]. Dunnet’s comparison’s test revealed that 3 mg/kg dFBr significantly reduced 20% ethanol intake [p<0.05] at the 4 hr time-point as compared to vehicle injected male rats. At the 24 hour time-point after dFBr administration, ANOVA found a significant difference in ethanol consumption among treatment groups [F (2.181, 15.27) = 6.757, p<0.00001]. ANOVA also found a significant difference in ethanol preference among treatment groups [F (2.116, 14.81) = 2.468, p<0.05]. Specifically, the 3 mg/kg dFBr dose significantly reduced preference for ethanol [p<0.05] at the 24 hr time-point as compared to vehicle injected male rats (Fig 2).

**Table 1:** Effects of vehicle/dFBr treatment on water and ethanol intake at 4 hr and 24 h time-point post injection in male and female SD rats consuming 20% ethanol in the IA2BC model.

**Males**				
	Water intake (g/kg/4 hr)	Water (g/kg/24 hr)	Ethanol (g/kg/4 hr)	Ethanol (g/kg/24 hr)
Vehicle	25.46 ± 2.76	43.38 ± 3.94	2.15 ± 0.28	5.84 ± 0.39
1 mg/kg dFBr	19.04 ± 1.78	47.03 ± 4.21	1.93 ± 0.32	4.95 ± 0.43*
3 mg/kg dFBr	23.88 ± 2.16	50.68 ± 3.12	1.52 ± 0.3*	4.08 ± 0.34****
1 week post-treatment	19.09 ± 1.66	47.20 ± 4.77	2.26 ± 0.32	6.21 ± 0.39
**Females**				
	Water intake (g/kg/4 hr)	Water (g/kg/24 hr)	Ethanol (g/kg/4 hr)	Ethanol (g/kg/24 hr)
Vehicle	43.63 ± 3.25	85.62 ± 6.19	1.93 ± 0.17	5.01 ± 0.35
1 mg/kg dFBr	38.08 ± 4.57	100.8 ± 7.15	0.99 ± 0.24*	3.1 ± 0.61**
3 mg/kg dFBr	51.55 ± 5.69	110.1 ± 9.07*	1.01 ± 0.19*	3.46 ± 0.56*
1 week post-treatment	39.96 ± 3.49	106.0 ± 8.13*	1.91 ± 0.24	4.93 ± 0.52

The values are expressed as mean fluid intake ± SEM (ANOVA followed by Dunnett’s multiple comparisons test).

*, *p<* 0.05

**p<0.01

**** *p*<0.0001, compared with vehicle-treated group. n = 23 males/group (experiment 1+2 combined) and 12 females/group (experiment 2) for 24 hr time-point. n = 8 males/group (experiment 2) and 12 females/group (experiment 2) for 4 hr time-point.

In female rats, dFBr showed similar decreases in ethanol intake and preference. At the 4 hour time-point, ANOVA found a significant difference in ethanol consumption among female treatment groups [F (2.714, 29.86) = 10.06]. Both 1 and 3 mg/kg dFBr reduced ethanol intake [p<0.05] at the 4 hr time-point as compared to vehicle injected female rats. In case of the 24 hr time-point, ANOVA found a significant difference between vehicle and dFBr treated rats (F (2.645, 29.09) = 6.459, p<0.01). Significant decreases in ethanol consumption at the 24 hr time-point were found with 1 mg/kg dFBr [p<0.01] and 3 mg/kg dFBr [p<0.05] in female rats. ANOVA found a significant difference between ethanol preference in vehicle vs. dFBr injected female rats at the 24 hour time-point [F (2.583, 28.42) = 6.371, p<0.01]. Both 1 and 3 mg/kg dFBr doses significantly reduced preference for ethanol [p<0.05] at the 24 hr time-point as compared to vehicle injected female rats (Fig 2). During the washout period of 1 week after the last dFBr injection, both male and female rats treated with dFBr did not show any increases in ethanol consumption and preference as compared to vehicle control rats. dFBr treatment also did not cause any significant alterations in body weight as shown in S1 Fig.

### dFBr does not alter sucrose consumption

**Fig 3:**
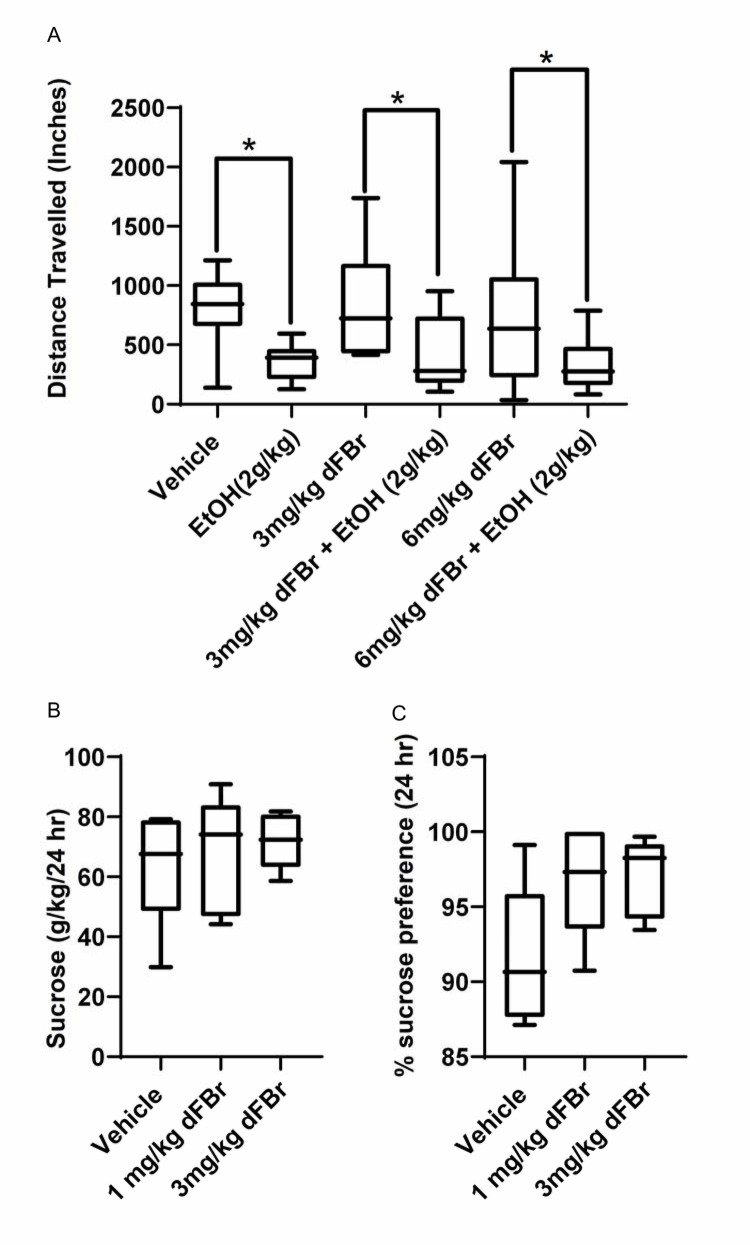
A: The distance traveled in an open-field arena was measured to test locomotor activity after injection of vehicle, 2 g/kg ethanol (EtOH), 3 or 6 mg/kg dFBr and 3 or 6 mg/kg dFBr+ 2 g/kg EtOH. n = 10–15 SD rats. 2g/kg EtOH significantly decreases locomotion in SD rats, while dFBr alone does not affect locomotion significantly in comparison to vehicle-treated rats. Pretreatment with dFBr does not alter the ethanol- induced decrease in locomotion. Data are represented as mean ± S.E.M. * *p*<0.05 compared to the vehicle treated group, n = 10 to 15/ treatment group. B. dFBr (1 and 3 mg/kg, 3 doses on Mon, Wed and Fri of Week 12) does not change sucrose consumption at the 24-hr time-point as compared to vehicle treated group, n = 8 rats/treatment group C. dFBr (1 and 3 mg/kg, 3 doses on Mon, Wed and Fri of Week 12) does not change % sucrose preference at the 24-hr time-point as compared to the vehicle treated group, n = 8 rats/treatment group.

In the first experiment with male rats, we also employed a parallel cohort of male rats which had intermittent access to 20% sucrose or water. dFBr injections (1 and 3 mg/kg) on Mon, Wed and Fri of Week 12 did not alter sucrose consumption or preference (Fig 3B and 3C) as compared to vehicle-treated rats.

### Locomotor activity

We studied the acute effect of dFBr administration on locomotor activity in an open field assay for up to 30 minutes after dFBr injection in male SD rats. Treatment with 3 and 6 mg/kg dFBr alone did not significantly alter the total distance traveled (Fig 3A). Unpublished data from our laboratory using the Novel Object Recognition assay (not shown here) suggest that the lower dose of 1 mg/kg dFBr does not cause any change in overall locomotor behavior in the arena. For the open field assay, we chose the doses of 3 and 6 mg/kg dFBr, in order to include a higher dose in investigating locomotor activity. In addition, the rationale for including the 6 mg/kg dose was our earlier observation that this dose of dFBr attenuated the effects of hypnotic dose of ethanol (4 g/kg) in a loss-of-righting reflex (LORR) assay, without altering blood alcohol levels [15]. Here, we studied the effects of low dose of ethanol (2 g/kg) alone and in rats pretreated with dFBr. ANOVA found a significant difference across various treatment groups in the open field assay [F (5, 64) = 4.893, p<0.001]. As expected, Bonferroni multiple comparisons test showed that low dose ethanol (2 g/kg) significantly decreased locomotor activity in male rats [p<0.05]. Neither 3 mg/kg nor 6mg/kg dFBr treatment significantly altered locomotor activity. Further, pretreatment with either 3 mg/kg or 6 mg/kg dFBr before administration of ethanol did not differ from the decreased locomotion seen after ethanol administration alone. As compared to dFBr treated animals, animals treated with dFBr+ ethanol showed significantly decreased locomotion [p<0.05], thus pretreatment with dFBr did not change the animals’ response to low-dose ethanol.

## Discussion

We have previously shown that dFBr decreased the response to a sedative-hypnotic dose of ethanol and attenuated acute ethanol-induced increase in the surface levels of the α_4_ nAChR subunit [15]. In this study, we show that dFBr (1 and 3 mg/kg) can also reduce ethanol intake and preference in the IA2BC model of voluntary ethanol consumption employing male and female SD rats. dFBr did not alter consumption of 20% sucrose or locomotion in parallel studies.

A number of genetic studies have studied the role of nAChR subtypes in alcohol drinking behavior. For example, acute alcohol drinking behavior and alcohol-induced midbrain dopaminergic function is reduced in α4 KO mice as compared to wild type (WT) suggesting the involvement of nAChR α4 subunit in alcohol abuse [10,21]. Conversely, β_2_ KO mice do not show alterations in alcohol drinking behavior as compared to WT type mice [22]. Thus, research indicates that brain nAChR subtypes are important mediators of the rewarding effects of ethanol, in addition to nicotine [23], suggesting that drugs targeting nAChR could be used for the treatment of alcohol and nicotine co-abuse, which occurs commonly. Mecamylamine, a non-selective nAChR antagonist reduces ethanol drinking in a number of animal models [24–26], with mixed results in human studies [27–29]. Furthermore, the selective α_4_β_2_ nAChR antagonist dihydro-β-erythroidine (DHβE) did not reduce ethanol intake [30]. These studies suggest mixed efficacy for treating ethanol dependence through nAChR blockade. Varenicline, a α_4_β_2_ nAChR partial agonist that is a FDA-approved smoking cessation aid [31], was found to reduce alcohol drinking in both animal models and humans [32–38]. However, the side effect profile of vareniciline such as nausea, vomiting, flatulence, constipation, weight gain, and dizziness, headache, and sleep disturbances appears to be significant enough to prompt premature discontinuation in adults trying to quit smoking [39].

Over the past few years, many α_4_β_2_ nAChR agonists aimed to improve cognitive functioning have not translated into clinically beneficial agents [40]. One explanation for the limited therapeutic efficacy of nAChR agonists is thought to be agonist-induced receptor desensitization. Due to the challenges associated with α_4_β_2_ nAChR agonists and antagonists as therapeutic candidates, nAChR subtype-selective positive allosteric modulators (PAMs) have emerged as a new focus. dFBr acts as a Type II PAM that potentiates maximal ACh currents [13] is thought to alter channel gating of recombinant α_4_β_2_ nAChR by increasing the channel opening frequency, prolonging open channel duration and shifting the equilibrium from the desensitized to open confirmation [41]. As a type II PAM, dFBr has no intrinsic activity, but instead increases the activity of an agonist acting at the target receptor and may not lead to tolerance, which is typically associated with agonists [16]. It is important to note that the electrophysiological characterization of dFBr thus far has been carried out on recombinant nAChR expressed in *Xenopus laevis* oocytes, which may differ from native nAChR. Functional characterization on native nAChR in brain slices could shed more light on the mechanisms by which dFBr modulates nAChR (effects on channel kinetics, desensitization etc). dFBr has been shown to decrease nicotine intake; while not substituting for nicotine in supporting self-administration, indicating a low liability for abuse and dependence [17]. dFBr also crosses the blood-brain barrier within 30 minutes of s.c. injection, with a predicted half-life of 8.6 hours [17]. These data, along with dFBr’s ability to attenuate the hypnotic effects of alcohol (previously reported by us in [15]), and the results with ethanol drinking reported here support the feasibility of further developing dFBr as a therapeutic candidate for AUD. Yet another nAchR ligand is Sazetidine-A, which does not activate α_4_β_2_ nAChR, but reported as a ‘silent desensitizer’ of α_4_β_2_ nAChR [42]. Sazetidine-A decreases alcohol and nicotine self-administration in alcohol preferring P rats [43]. In contrast, another report found that Sazetidine-A can in fact activate α_4_(2)β_2_(3) and α_4_(3)β_2_(2) stoichiometries of nAChRs with varying efficacies and also stimulate nAChR-mediated dopamine release from striatal slices [44]. These findings support the hypothesis that drugs that activate α_4_β_2_ nAChR maybe useful in attenuating alcohol and nicotine dependence, albeit dFBr activates α_4_β_2_ nAChR via the PAM mechanism.

We employed an IA2BC model that has been previously employed by various groups [20]. Similar to previous reports [35,45,46], we found that male rats escalated drinking to ~6 g/kg/24 hr in the 11 week timeline. To our knowledge, these previous reports and others have not employed female SD rats in a similar IA2BC paradigm. We found that female rats did not significantly escalate their ethanol drinking up to 9 weeks into the IA2BC timeline, but rather maintained levels of ~5g/kg/24 hr from week 1–9. Comparing male and female counterparts in the experiment 2, we found that male SD rats consumed more ethanol (g/kg body weight) and showed higher preference for ethanol as compared to female rats. Previous two-bottle choice test studies have reported that females tend to drink more than males and show higher preference for alcohol over water. However, unlike our study which employed SD rats, many of these prior studies have employed Wistar/Long Evans rats or inbred lines of mice/rats. Furthermore, these studies greatly vary in the type of alcohol presented (% of ethanol, beer/sucrose mixed with ethanol etc.), duration of alcohol availability (continuous/ intermittent/ 30 min sessions etc.) and single versus group housing [47–54]. Moreover, some studies have reported equal consumption between males and female P and CD rats [55,56]. This disparity may again be related to animal strain differences; and strain is recognized as an important determining factor of voluntary alcohol consumption in both rats and mice [47,51].

Among studies that employed SD rats, it has been shown that adolescent males consume more alcohol relative to their body weights than adolescent females as well as adults of both genders [57]. The same study also found that adult females consumed more ethanol than adult males [57]. However, we note that this said study in SD rats employed a 2 hr access to ethanol for 8 days as compared to the ~11 week IA2BC model employed in our study. In another study with SD rats, adolescent rats consumed significantly higher amounts of an ethanol-Boost® solution as compared to adults, however no sex-based difference was seen in ethanol consumption in adult rats [58]. Thus, our finding that adult female SD rats show lower ethanol consumption and preference as compared to adult males could be attributed to several factors including strain, study paradigm, type of alcohol presented etc. We chose SD rats for our study since our earlier study focused on dFBr’s effect on the hypnotic response to ethanol was carried out in this strain [15]. It is important to note that the IA2BC model in SD rats has limitations such as inherent variability in drinking levels (such as ‘drinkers’ and ‘non-drinkers [59]). Similarly, we observed lower % ethanol preference values (ranging from 5–34% in males) in agreement with ‘preferrers’ and ‘non-preferrers’ reported earlier in SD males [60]. We did not divide the population into separate groups, but we did exclude 2 male rats/group from dFBr administration since they did not significantly escalate their drinking levels over 11 weeks as compared to other males in the same cohort. It has been suggested that the variability seen with outbred SD rats contributes to the validity of the model since it reflects individual differences seen in heterogeneous human populations [61]. For example, similar to the gender-based differences observed by us, we know that women drink less compared to men [62].

Our results suggest that dFBr (3 total doses administered once per day on Mon, Wed and Fri after establishment of IA2BC) significantly decreases ethanol consumption and preference in both male and female rats at the 4 and 24 hr time-points. We also did not observe a rebound increase in ethanol consumption after a 1 week washout period. However, the half-life of dFBr in rats is ~ 8.6 hrs, suggesting that a greater reduction in ethanol consumption may be seen if dFBr is administered twice a day in animals. The half-life, safety and efficacy profile of dFBr in humans remains to be elucidated. Moreover, it is recognized that the rat model can play an important role in providing reasonably accurate prediction of human half-life [63]. Further pharmacokinetics studies are needed to predict half-life of dFBr in humans.

Our findings suggest that dFBr administration does not significantly alter locomotion. In addition, pretreatment with dFBr also does not alter the decrease in locomotion seen with a low dose of ethanol. The data from both male and female rats in the IA2BC test also suggest that dFBr likely does not affect locomotion since dFBr reduced ethanol consumption, without reducing water consumption. In fact, dFBr significantly increased water consumption at the 24 hr time-point (female rats). Previously, we reported that dFBr counteracts sedative-hypnotic effects after a single intoxicating dose of ethanol, likely acting via the β_2_ nAChR subunit [15]. We also found that a single intoxicating dose of ethanol causes an early increase in thalamic α_4_ nAChR subunit levels and that dFBr attenuates this ethanol-induced increase in the α_4_ nAChR subunit. Previous data also suggest that dFBr can decrease compulsive-like behavior, neuropathic pain, and nicotine self-administration [17–19]. Since nicotine and ethanol are commonly co-abused and may act via common pathways in the reward pathway, a drug that can target both drugs of abuse is desirable. It has been suggested that dFBr, acting as a α_4_β_2_ nAChR PAM may reduce the amount of nicotine self-administered via smoking since smokers treated with dFBr may consume less nicotine while deriving the same degree of the subjective reinforcing effects of nicotine [17]. In order to test the effects of dFBr on both ethanol and nicotine consumption, dFBr will need to be further evaluated in appropriate models that can examine consumption of both these substances. In order to further test its utility as a drug for treatment of alcohol use disorder, dFBr needs to be further evaluated on animals models of high alcohol consumption (such as P rats and the UChB rats) and on alcohol self-administration models in order to understand the motivational and reinforcement aspects of alcohol drinking behaviors.

## Supporting information

S1 Fig S1A Fig shows body weight of male rats after treatment with vehicle or dFBr and 1 week post-treatment. S1B Fig shows body weight of female rats after treatment with vehicle or dFBr and 1 week post-treatment.
(PDF)
