# Supplementary figures and images for: Desformylflustrabromine (dFBr), a positive allosteric modulator of α4β2 nicotinic acetylcholine receptors decreases voluntary ethanol consumption and preference in male and female Sprague-Dawley rats
Source: PLoS One. 2022 Sep 9;17(9):e0273715. doi: 10.1371/journal.pone.0273715 (PMC9462806; doi:10.1371/journal.pone.0273715)

Supplementary Fig 1

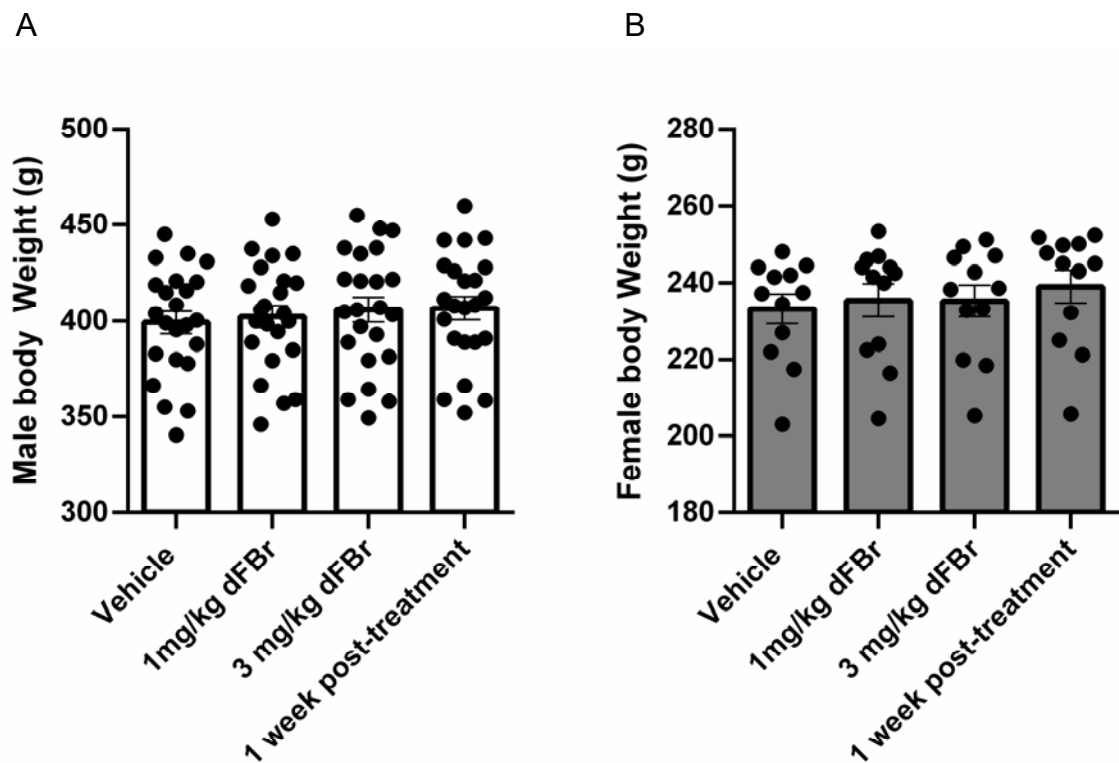

Supplement: S1 Fig — S1A Fig shows body weight of male rats after treatment with vehicle or dFBr and 1 week post-treatment. S1B Fig shows body weight of female rats after treatment with vehicle or dFBr and 1 week post-treatment. (PDF) [file pone.0273715.s001.pdf]
